# Protective effect of Pinacidil on hypoxic-reoxygenated cardiomyocytes *in vitro* and *in vivo* via HIF-1α/HRE pathway

**DOI:** 10.1371/journal.pone.0318859

**Published:** 2025-02-24

**Authors:** Ying Wang, Jiaqi Wang, Liangen Chen, Wenjing Zhou, Haifeng He, Xiyuan Chen, Haiying Wang

**Affiliations:** 1 Department of Anesthesiology, Affiliated Hospital of Zunyi Medical University, Zunyi, Guizhou, China; 2 School of Anesthesiology, Zunyi Medical University, Zunyi, Guizhou, China; Georgia State University, UNITED STATES OF AMERICA

## Abstract

Cardiomyocyte hypoxia-reoxygenation (HR) is considered as a major cause of heart failure. Pinacidil is a classic ATP sensitive potassium channel opener and plays a crucial role in cardiomyocyte HR injuries. However, the specific mechanism is poorly understood. We established HR rat model and introduced 5-Hydroxydecanoate (5-HD), N-(2-Mercaptopropionyl)-glycine (MPG), and Dimethylethylenediylglycine (DMOG) to investigate the protection of Pinacidil (P) on cardiomyocyte. HE staining, electron microscopy and JC-1 staining were used to observe mitochondrial structure and mitochondrial membrane potential (MMP). Reactive oxygen species (ROS), hypoxia-inducible factor-1α (HIF-1α), vascular endothelial growth factor A (VEGF-A), heme oxygenase-1 (HO-1), and induced nitric oxide synthase (iNOS) were analyzed in this study. Network pharmacology analysis and auto-docking were used to predict the possible target of Pinadicil under cardiomyocyte HR condition. The integrity of mitochondrial structure and MMP were effectively promoted in P and MPG+DMOG + P groups. ROS was significantly increased after HR, treatment with P or MPG+DMOG + P, the content of ROS was increased. The expressions of HIF-1α, VEGF-A, HO-1 and iNOS were significantly increased in P and MPG+DMOG + P groups compared with HR group. Docking results confirmed that prolyl hydroxylase (PHD) was the most possible target for unsaturated binding with Pinacidil guanidine. Altogether, these data indicate that Pinacidil up-regulated and activated HIF-1α protein to protect caridomyocytes against HR injuries and the mechanism may be related to Pinacidil guanidine binding to PHD.

## Introduction

Ischemia-reperfusion (IR) injury is the most common reason of cardiomyocytes death, which eventually progresses to heart failure [1]. Cardiac ischemia de-oxygenates the myocardium, and reperfusion re-introduces oxygen to the myocardium to induce marked hyper-oxygenation injury, which is characterized by mitochondrial dysfunction, excess reactive oxygen species (ROS) production, and cell death [2,3]. Increasing evidences indicate that hypoxia-inducible factor (HIF) [4] and ATP-sensitive potassium (KATP) channel [5] in mitochondria are the most common forms of cellular response to oxidative injury during hypoxia-reoxygenation (HR). HIF is a heterodimer transcription factor in nuclear, which is an essential hypoxia-response protein that facilitates the transcription of hypoxia-responsive genes under hypoxia condition [6,7].

Structurally, one of three oxygen-regulated α-subunits (HIF-1α, HIF-2α, and HIF-3α) and a constitutively expressed β-subunit (HIF-1β) compose the dimer transcription factor HIF. Among them, HIF-1α plays an essential role in regulator for oxygen concentration in tumor proliferation, endothelial cells response, and cardiomyocyte hypoxia [8,9]. The stability of HIF-1α is dependent on prolyl hydroxylase domain (PHD), which plays an important role in many ischemic disorders including myocardial infarction, peripheral arterial occlusive disease, and stroke [10]. The hypoxia-responsive genes including erythropoietin (EPO), vascular endothelial growth factor-A (VEGF-A), and glycolytic enzymes, which are regulated by transcription factors HIF, are induced in hypoxia conditions [11]. HIF binding to hypoxia-responsive elements (HRE) induce transcription of downstream targets: VEGF, heme oxygenase-1 (HO-1), induced nitric oxide synthase (iNOS) in cardiomyocyte [12]. Previous reports revealed that amphiregulin participates in the inhibition of myocardial ischemia-reperfusion injury through HIF-2α dependence pathway [13,14]. However, HIF-1α/HRE pathway has been a largely under explored domain in HR cardiomyocytes.

Pinacidil has been widely known as an ATP-sensitive potassium channel (KATP) opener that has notably ability to drive ischemic myocardium protection and exerts profound cardioprotective effects through regulation Ca^2 +^ related receptors [15–18]. It has been reported that Pinacidil protected cardiomyocytes via mitochondrial KATP channels in a rat model of cardiac ischemia/reperfusion [19]. However, the possible mechanisms of Pinacidil protecting ischemia-reperfusion cardiomyocytes are still unclear. Therefore, we will investigate the cardioprotection of Pinacidil through HR rat model, and further explore the potential mechanism of Pinacidil through both in vivo and in vitro models of hypoxia or HR damage.

## Materials and methods

### Animal

The animal experiments of this study were performed in accordance with the Guide for the Care and Use of Laboratory Animals in China and were supported by Experimental Animal Care and Use Committee of Zunyi Medical University (No. SCXK 2019-0010). The rats were anesthetized by intraperitoneal injection of sodium pentobarbital (40 mg/kg) and then were sacrificed at the experimental endpoint.

Fifteen-week-old male Sprague-Dawley (SD) rats (250–300 g) were acquired from Changsha Tianqin Biotechnology (http://cstqsw.com) and were housed in a temperature-controlled room with a 12:12-h light-dark cycle. Animals were providing free access to standard laboratory chow and clean water.

### Isolation of adult rat cardiomyocytes and establishment of ischemia-reperfusion rat heart model

**Fig 1:**
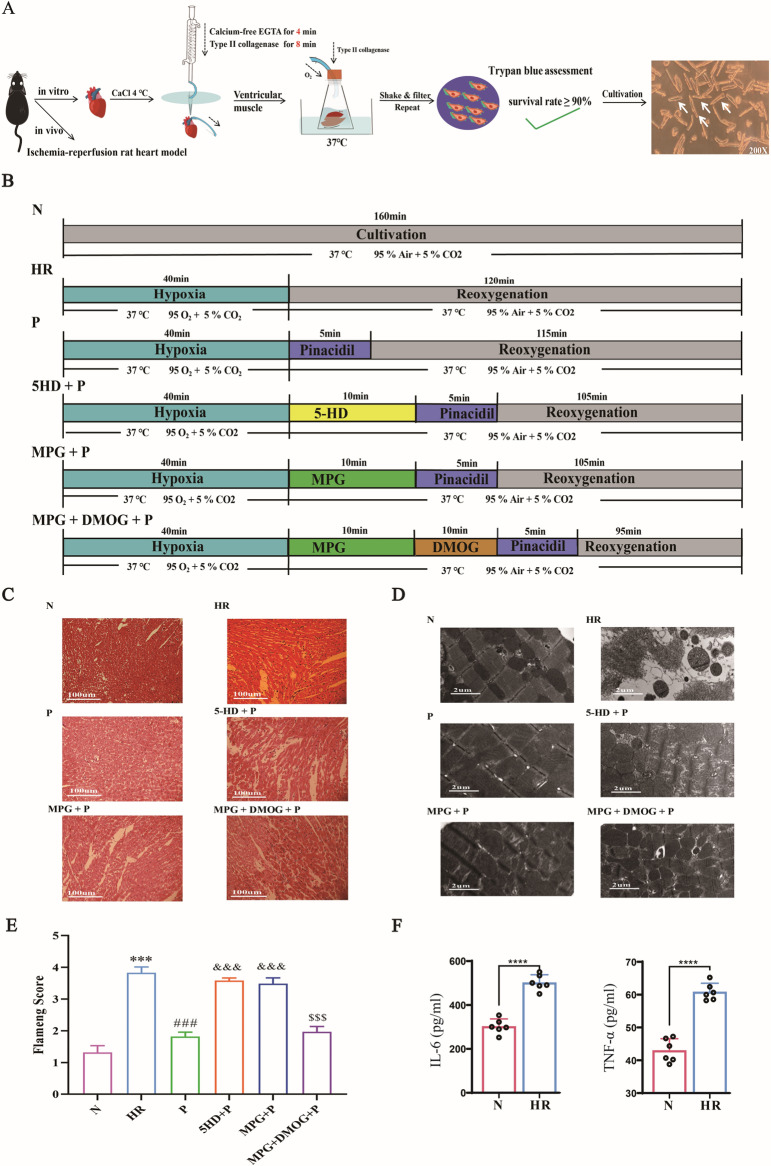
Schematic of experimental design and Pinacidil improves the HR-induced cardiomyocyte structure injury *in vivo.* (A) Experimental design chart; (B) Experimental grouping and interventions; (C) Rat heart tissue stained with hematoxylin and eosin; (D) Ultrastructure of cardiomyocyte under electron microscopy; (E) Flameng scores; (F) Levels of IL-6 and TNF-α. (mean ±  SD), ***P < 0.001, ****P < 0.0001 *vs* N group; ###*P* < 0.001 *vs* HR group; &&&*P* < 0.001 *vs* P group; $$$*P* < 0.001 *vs* MPG + P group.

The rats were intraperitoneally injected with sodium pentobarbital (40 mg/kg) and heparin (250 g/kg). After the rats were anesthetized, their skin was disinfected with alcohol, and then the hearts were extracted to isolate rat cardiomyocytes. The ventricular cardiomyocytes were obtained by enzyme digestion. Briefly, the rat heart was washed in 4 °C to remove the blood. And then the heart was pumped with fully oxygenated calcium solution (750 μM) at the flow rate (9 mL/min/g), then successively perfused with calcium-free EGTA solution (100 μmol/L) for 4 min, and type II collagenase for 8 min by Langendorff instrument. After infusion, the heart was cut into 3–4 pieces and sank in 2 mL type II collagenase containing 2% BSA, shook and oxygenated in 37 °C for 5 min. After that, the above tissue solution was filtered by a sterile 200-mesh nylon filter in to a new sterile tube and placed it for precipitation. The above steps were repeated 5 times. All tubes precipitation was collected. Isolated cells were cultured in the dish pre-coated with laminin by modified M199 medium (with 2 mM carnitine and glutamine, 5 mM taurine and creatine, 0.8 mM EGTA). After 4 h incubation, the medium was changed to remove the non-cardiomyocytes, and the surviving cells were determined by Trypan blue. The schedule of this study is shown in Fig 1A.

The rat heart ischemia-reperfusion model was established according to the reference [20] in this study. After anesthetized, the rat pericardium was opened to expose the heart. The origin of left anterior descending coronary artery (LAD) was occluded with a suture (6.0 nylon) for 30 min, and then the ligature was removed to allow reperfusion. The wound was sutured and the rats were injected with buprenorphine (0.05 mg/kg, SQ). During the recovery period, the animals received supportive post-operative care as needed. The body temperature was maintained at 37 °C with a heat pad. After 12-h post-operation, Pinacidil, 5-Hydroxydecanoate (5-HD, a selective KATP channel blocker), N-2-Mercaptopropionyl-glycine (MPG, a synthetic aminothiol antioxidant) and Dimethyloxallyl Glycine (DMOG, a hypoxia-inducible factor prolylhydroxylase inhibitor) were injected into tail vein [17]. Another 12 h, the rats were anesthetized and decapitated to collect the hearts.

### Preparation and treatment of hypoxic cardiomyocytes

As shown in Fig 1B, cardiomyocytes were incubated (95% air + 5% CO_2_, 37 °C) for 24 h and randomly divided into 6 groups: normal group (N): incubated with 95% air + 5% CO_2_ for 160 min at 37 °C in an incubator. Hypoxia group (HR): incubated in a 95% N_2_ + 5% CO_2_ hypoxia chamber for 40 min at 37 °C, and then incubated in a reoxygenation chamber (95% air + 5% CO_2_) for 120 min. Pinacidil treatment group (P): after 40 min hypoxia treatment, Pinacidil (50 μmol/L [12]) was added and the cardiomyocytes were incubated for another 5 min. 5-HD + P group, MPG + P group and MPG +  DMOG +  P group: 5-HD (100 μmol/L [21]), MPG (2 mmol/L [17]), or MPG +  DMOG (100 μmol/L [17]) pre-treated the cells for 10 min, and then Pinacidil was added at the beginning of reoxygenation.

### Detection of cell viability, inflammatory cytokines and ROS level

The cell viability was measured by MTT assay according to the manufacture's instruction. The ELISA kits were used to test the levels of cytokines interleukin-6 (IL-6) and tumor necrosis factor α (TNF-α) (Shanghai Hengfei Biotechnology Co., LTD., Shanghai, China), as well as ROS (R&D Systems) at 25 min and 120 min after reoxygenation.

### H&E staining and Electron microscopy analysis

Formalin-fixed, paraffin-embedded tissue slices of 10 μm were cut on glass slides for morphological detection. Hematoxylin and eosin staining (H&E) (Changsha Abiowell Biotechnology Co., China) was used to define the tissue integrity and myocardial fibrosis.

The cells were fixed in 2.5% glutaraldehyde at 4 °C for 2 h, and then gradient dehydration, embedding polymerization, ultra-thin slices, double stained with uranyl acetate and lead citrate, the slices were observed by electron microscopy (Japan, Hitachi H7500). Five fields were selected in each specimen, and 20 mitochondria were selected from each field, and each mitochondrion was scored at 10,000 times magnification based on the Flameng scoring criteria [22] (Table 1). Higher scores represent worse injury in mitochondria.

**Table 1:** Flameng scoring criteria.

Score	Mitochondrial morphology
0	Normal structure, filled with particles
1	Normal structure, loss of matrix particles
2	Mitochondria swollen, matrix hyaline
3	Mitochondrial cristae fracture, matrix hyaline and condensation
4	Mitochondrial cristae split, loss of membrane integrity, vacuolation

### Mitochondrial membrane potential (MMP) measurement

The MMP of cardiomyocyte was determined by JC-1 (mitochondrial membrane potential dye) method, according to the kit instructions (Shanghai Byotime Bioteh, Shanghai, China), and observed under confocal laser microscope. JC-1 monomer was detected with excitation light of 514 nm and emission wavelength of 529 nm. For the detection of JC-1 polymer, the excitation light was 585 nm and the emission wavelength was 590 nm. The ratio of red to green fluorescence in each cell was determined using the Image Pro Plus system.

### Real-time quantitative PCR

Total RNA was collected with TRIzol protocol using TaKaRa RNAiso Kit (TaKaRa, Japan). RNA concentration and purity were checked using Varioskan Flash spectrophotometer (Thermo Fisher, Waltham, MA, USA). According to cDNA synthesis kit (TaKaRa, Shiga, Japan), 500 ng RNA was reverse transcribed into cDNA at 37 °C for 15 min and 85 °C for 5 s. Real-time quantitative PCR (RT-QPCR) was used to detect the expression of HIF-1α, HO-1, iNOS and VEGF mRNA using SYBR green PrimScript RT kit (TaKaRa, Shiga, Japan). GAPDH was selected as the internal reference gene. The primer sequences of these genes were shown in Table 2. Pre-degeneration (95 °C 30 s), cyclic reaction (95 °C 5 s; 55 °C 30 s) for 40 cycles and lysis reactions (55 °C for 30 s each time increasing by 0.5 °C to maintain) were performed on a PCR instrument. The 2^−ΔΔCt^ value of each group was calculated, which was the relative expression of genes in each group.

**Table 2.** The PCR primer sequences.

Gene	Forward (5’-3’)	Reverse (5’-3’)
HIF-1α	GGTGCTAACAGATGATGGTGAC	GGCTCATAACCCATCAAC
HO-1	CGGCCCTGGAAGAGGAGATAG	GGTGGGGTTGTCGATGCTCGG
iNOS	GAGGCCCAGGAGGAGAGAGATTC	GTAGCAAAGAGGACTGTGGCT
VEGF	CAATGATGAAGCCCTGGAGTG	GTCTGCGGATCTTGGACAAAC
GAPDH	ACAGCAACAGGGTGGTGGAC	TTTGAGGGTGCAGCGAACTT

### Western blot

Cardiomyocytes were lysed in RIPA buffer containing PMSF and protein phosphatase inhibitors, and subjected to western blot analysis. Anti-HIF-1α (20960-1-AP), anti-VEGF (19003-1-AP), anti-HO-1 (10701-1-AP), anti- iNOS (22226-1-AP), and anti- GAPDH (60004-1-Ig) antibodies were purchased from Proteintech (Wuhan, China). All proteins were separated by SDS-PAGE and transferred onto PVDF membranes. Finally, ECL was used to color the bands, which were exposed using a gel imaging system (Bio-Rad Laboratory, lnc.). Bands intensities were quantified using Image J software (NIH, USA), and GAPDH was used as an internal control.

### Molecular docking

Three-dimensional (3 d) structure of Pinacidil, HIF-1 alpha, HO-1, VEGF and iNOS was downloaded from https://zinc.docking.org/ and https://pubchem.ncbi.nlm.nih.gov/, respectively. The advantageous conformation of Pinacidil was generated by the DS ViewerPro Trial. Import these files into AutoDockTools-1.5.6 software, add hydrogen atoms and remove water. After 50 times of automatic docking, the results were displayed and analyzed using PyMOL software, Discovery studio 3.5 Client software and R 4.0.

### Statistical analysis

SPSS (v.22, IBM, USA) was used for the statistical analysis. All data were presented as mean ± SD, with *P* < 0.05 considered statistically significant. Differences between two groups were analyzed using Student’s t test, among multiple groups, one-way ANOVA followed by *Bonferroni* multiple comparison was used in this study.

## Results

### Pinacidil protects mitochondrial structure injury *in vivo* and *in vitro*

HR destroyed the cell structure evidenced by loss of cell integrity and intracellular partial lysis of myofibrils observed by H&E staining and electric microscope (Fig 1C and 1D). After Pinacidil treatment, the structure of cells in the P group was basically normal, the myofibrils in the matrix were neatly arranged, the Z- and H-lines were clear, and the myonodules could be distinguished as dark and light, similar to the normal cells in the N group. However, the effects of Pinacidil on cell structure were weakened by either MPG or 5-HD. DMOG could reverse the inhibitory effect of MPG on Pinacidil. Flameng score results showed that the score in HR group was higher than that in N group (t = −15.524, *P* < 0.001); the difference of Flameng score in the other groups was statistically significant (F[4,10] = 116.809, *P* < 0.001) (Fig 1E). Compared with HR group, Pinacidil treatment significantly decreased the score (*P* < 0.001), indicating that Pinacidil could protect cardiomyocyte against HR-induced injury. However, MPG or 5-HD suppressed the protective effect of Pinacidil on HR-induced myocardial injury (*P* < 0.001), and DMOG partially reversed the inhibitory effect of MPG on Pinacidil (*P* < 0.001).

Considering that IL-6 and TNF-α are often co-produced with ROS under hypoxia conditions, they represent the level of HR-induced cardiomyocytes damage [23]. Therefore, we tested the level of IL-6 and TNF-α, and the results showed in Fig 1F. The level of IL-6 and TNF-α were sharply increased in HR-induced cardiomyocytes compared with normal cardiomyocytes (TNF-α: t = 11.23, *P* < 0.001; IL-6: t = 7.57, *P* < 0.001), indicating that the HR model was successfully established.

**Fig 2:**
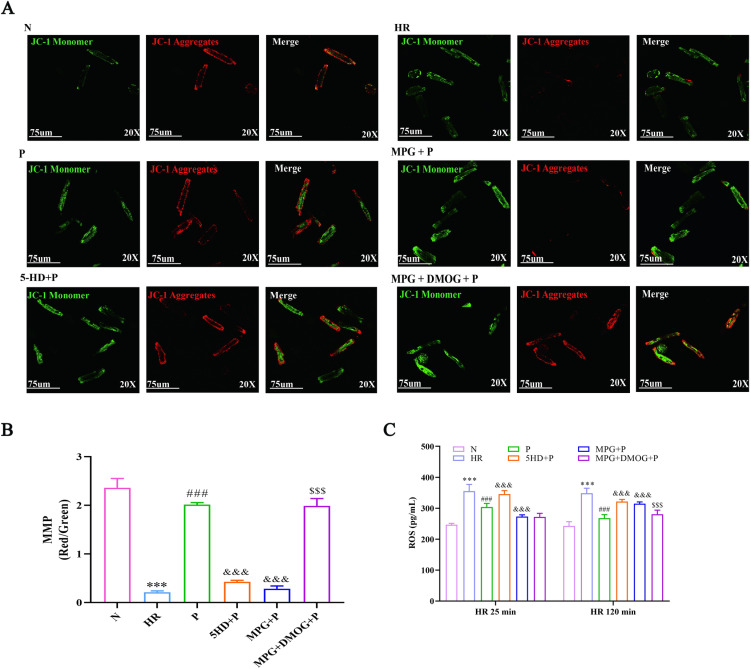
Pinacidil restores the MMP and ROS level in HR-induced rat cardiomyocytes *in vivtro.* (A) Representative images of MMP dyed by JC-1; (B) Quantitative analysis of MMP using the relative fluorescence intensity; (C) Quantitative analysis of ROS concentration. (n = 6, mean ±  SD), ***P < 0.001 *vs* N group; ###*P* < 0.001 *vs* HR group; &&&*P* < 0.001 *vs* P group; $$$*P* < 0.001 *vs* MPG + P group.

Mitochondrial membrane potential (MMP) reflects the healthy function of mitochondria [24]. The loss of MMP and the obstruction of ATP production are manifestations of mitochondrial dysfunction [25]. We detected the MMP level by JC-1 dye, and the results showed that JC-1 was mostly aggregated (red) in group N, by contrast, they had hardly aggregated and existed as monomer (green) form in HR-induced cardiomyocyte mitochondria (Fig 2A). Followed Pinacidil treatment, JC-1 aggregates (red) were increased, however, MPG or 5-HD inhibited the effect of Pinacidil on MMP. DMOG partially reversed the suppressive effect of MPG on Pinacidil. The ratio of JC-1 aggregator (red) and JC-1 monomer (green) was used to represent the level of MMP in each groups. Compared with N group, the ratio was sharply declined in HR (t = 19.972, *P* < 0.001). There was significant difference in MMP level between HR group and treatment groups (F[4,10] = 459.085, *P* < 0.001). Pinacidil increased the level of MMP versus HR group (*P* < 0.001), MPG or 5-HD suppressed the level of MMP increased by Pinacidil (*P* < 0.001), the MMP level in MPG+DMOG + P group was higher than that in MPG + P group (*P* < 0.001).

Next, we demonstrated the mitochondrial ROS generation after HR 25 min and 120 min, and the results were showed in Fig 2C that the ROS concentration was highly increased in HR group compared with N group at both 25 min (t = −11.630, *P* < 0.001) and 120 min (t = −15.656, *P* < 0.001). The different of ROS level was found among the other five groups at 25 min (F[4,18] = 30.024, *P* < 0.001) and 120 min (F[4,31] = 66.481, *P* < 0.001). After Pinacidil treatment, the ROS generation was significantly decreased at both HR 25 min and 120 min (*P* < 0.001); 5-HD counteracted the decreasing effect of Pinacidil on ROS generation at HR 25 min (*P* = 0.001) and 120 min (*P* < 0.001); however, MPG enhanced the decreasing effect of Pinacidil on ROS generation at HR 25 min (*P* = 0.013), but inhibited the effect of Pinacidil at HR 120 min (*P* < 0.001). DMOG+MPG + P did not show any significant difference at HR 25 min versus MPG + P group (*P* = 1.000), but showed the inhibitory effect of MPG at HR 120 min (*P* = 0.008).

### Pinacidil treatment activates the HIF-1α/HRE pathway in HR rat cardiomyocytes

**Fig 3:**
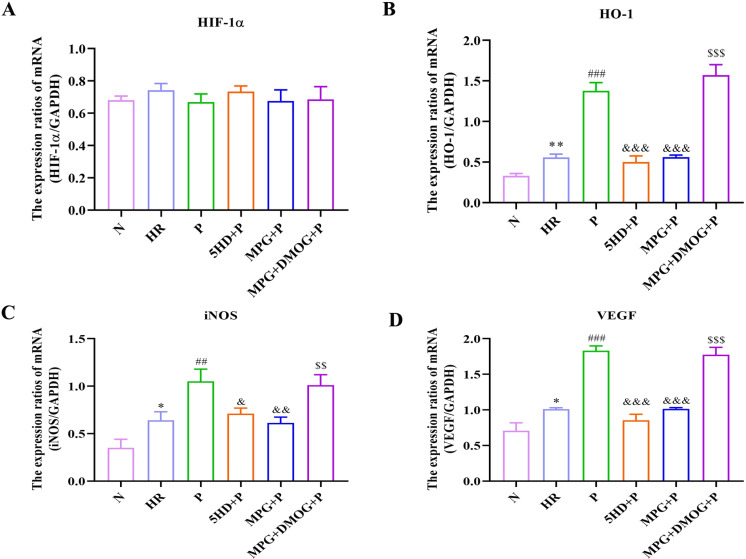
Effect of Pincacidil on the mRNA expressions of HIF-1 **α,** **VEGF, HO-1 and iNOS *in vitro*.** Quantitative analysis of mRNA expression of (A) HIF-1α; (B) HO-1; (C) iNOS; (D) VEGF by RT-qPCR (Normalized to GAPDH). (mean ±  SD), * *P* < 0.05, **P < 0.01, ***P < 0.001 *vs* N group; ##*P* < 0.01, ###*P* < 0.001 *vs* HR group; &*P* < 0.05, &&*P* < 0.01, &&&*P* < 0.001 *vs* P group; $$*P* < 0.01, $$$*P* < 0.001 *vs* MPG + P group.

According to that HIF-1α is translocated into mitochondria to defense against oxidative stress under hypo-oxygen conditions, we tested the mRNA and protein of the key molecules (e.g., HIF-1α, HO-1, iNOS, VEGF) in HIF-1α signaling pathway. The mRNA expression of HIF-1α was no significant difference among the groups (N group *vs* HR group: t = −2.313, *P* = 0.082; ANOVA of the other five groups: F[4,10] = 1.074, *P* = 0.419) (Fig 3A). While the mRNA levels of HO-1 (t = −7.800, *P* = 0.001), iNOS (t = −3.946, *P* = 0.017) and VEGF (t = −4.648, *P* = 0.010) were increased in HR group compared with N group (Fig 3B–D). The differences of the mRNA levels of HO-1 (F[4,10] = 115.064, *P* < 0.001), iNOS (F[4,10] = 14.772, *P* < 0.001) and VEGF (F[4,10] = 136.887, *P* < 0.001) among HR, P, SHD + P, MPG + P, MPG+DMOG + P groups were statistically significant. Compared with HR group, Pinacidil treatment largely enhanced the mRNA expression of HO-1 (*P* < 0.001), iNOS (*P* = 0.003) and VEGF (*P* < 0.001). 5-HD and MPG had inhibited the effect of Pinacidil on the mRNA expression of HO-1 (*P* < 0.001), iNOS (5-HD: *P* = 0.013; MPG: *P* = 0.002) and VEGF (*P* < 0.001). DMOG partially reversed the suppressed effect of MPG on Pinacidil (HO-1: *P* < 0.001; iNOS: *P* = 0.004; VEGF: *P* < 0.001) (Fig 3B–D).

**Fig 4:**
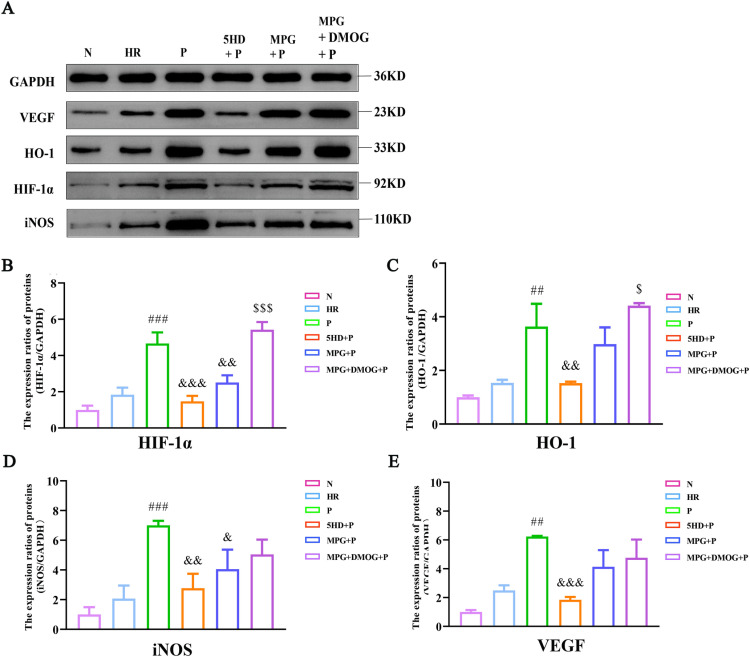
Pincacidil enhances the protein expression of HIF-1 **α,** **VEGF, HO-1 and iNOS *in vitro*.** (A) Representative bands stained by Western blot; Quantitative analysis of protein expression of (B) HIF-1α; (C) HO-1; (D) iNOS; (E) VEGF (Normalized to GAPDH). (mean ±  SD), ##*P* < 0.01, ###*P* < 0.001 *vs* HR group; &*P* < 0.05, &&*P* < 0.01, &&&*P* < 0.001 *vs* P group; $*P* < 0.05, $$$*P* < 0.001 *vs* MPG + P group.

Similarly, the protein expression of HIF-1α (F[5,12] = 57.826, P < 0.001), HO-1 (F[5,12] = 29.456, P < 0.001), iNOS (F[5,12] = 17.692, P < 0.001) and VEGF (F[5,12] = 22.348, P < 0.001) were statistically significant among groups (Fig 4). The abovementioned proteins expressions were increased in HR group compared with N group. Compared with HR group, the abovementioned molecules were significantly elevated after Pinacidil treatment. Similar to the aforesaid parameters, 5-HD and MPG had inhibited the effect of Pinacidil, DMOG partially reversed the suppressed effect of MPG on Pinacidil (Fig 4).

### PHD works as the main potential target of Pinacidil

**Fig 5:**
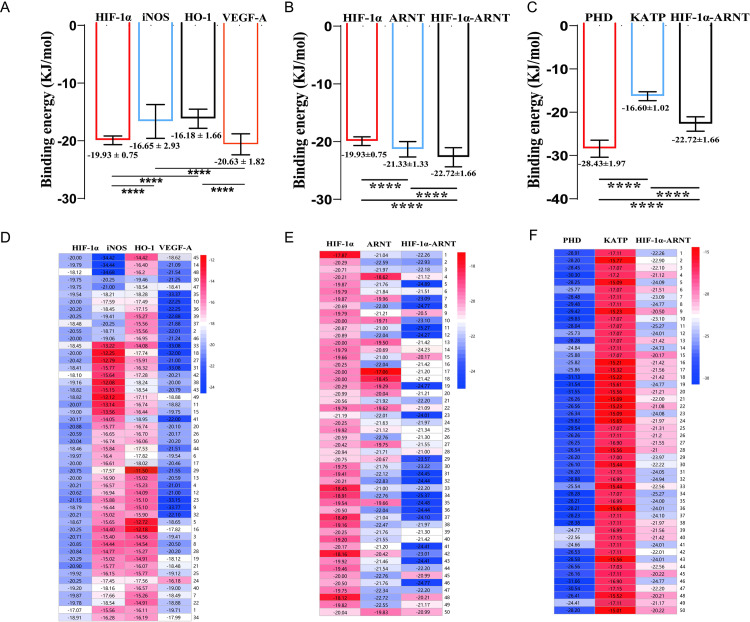
Pincacidil tends to target with HIF-1 **α,** **iNOS, HO-1, VEGF-A, ARNT, HIF-1α-ARNT, KATP and PHD.** (A-C) Quantitative analysis of binding energy; (D-F) Heatmap of binding energy distribution. Data are presented as mean ±  SD (n = 50). ****P < 0.0001.

To determine the pharmacological targets of Pinacidil, we introduced the Autodocking method to predict molecular docking between Pinacidil and HIF-1α/HRE pathway molecules. Normally, the binding free energy ( < −20.92 KJ/mol) indicates that the docking molecule has good binding activity with the target [26]. The results of Fig 5A and D showed that the binding energy of Pinacidil with HIF-1α was −19.93 ± 0.75, iNOS was −16.65 ± 2.93, HO-1 was −16.18 ± 1.66, VEGF-A was −20.63 ± 1.82. And the binding energy was significantly different of HIF-1α compared with iNOS (*P* < 0.0001); or HO-1 (*P* < 0.0001).

Under hypoxic conditions, HIFs will bind to ARNT (also known as HIF-1β), acting as the transcription factor of HRE [27]. In order to explore whether the HIF-1α-ARNT complex plays a role in Pinacidil pharmacology, we further observed the binding energy of ARNT or HIF-1α-ARNT complex with Pinacidil (Fig 5B and E). The results showed that the binding energy of Pinacidil with ARNT was −21.33 ± 1.33, with HIF-1α-ARNT complex was −22.72 ± 1.66, and the significant difference was found among them (ARNT *vs.* HIF-1α, *P* < 0.0001; HIF-1α-ARNT *vs*. HIF-1α, *P* < 0.0001; HIF-1α-ARNT *vs*. ARNT, *P* <  0.0001). It has been reported that HIF-1α can be up-regulated by HIF-1α prolyl hydroxylase (PHD) [28]. As well Pinacidil is widely known as ATP-sensitive potassium channel (KATP) opener [29]. On this basis, we observed the binding of Pinacidil and PHD or KATP, the results showed (Fig 5C and F) that the propensity target potent of PHD to Pinacidil was -28.43 ± 1.98, and KATP was 16.31 ± 1.02, (PHD *vs*. HIF-1α-ARNT, *P* < 0.0001; KATP *vs*. HIF-1α-ARNT, *P* < 0.0001; PHD *vs*. KATP, *P* < 0.0001).

In summary, HIF-1α, iNOS, HO-1, VEGF-A, ARNT, HIF-1α-ARNT, PHD and KATP can naturally act with Pinacidil. However, among these molecules, only PHD (binding energy distribution concentrated in −27.5 ~  −28.5 kJ/mol) has the strongest binding energy, suggesting that PHD may be the most possible target of Pinadicil.

### Guanidine is the key pharmacological group of Pinacidil

**Fig 6:**
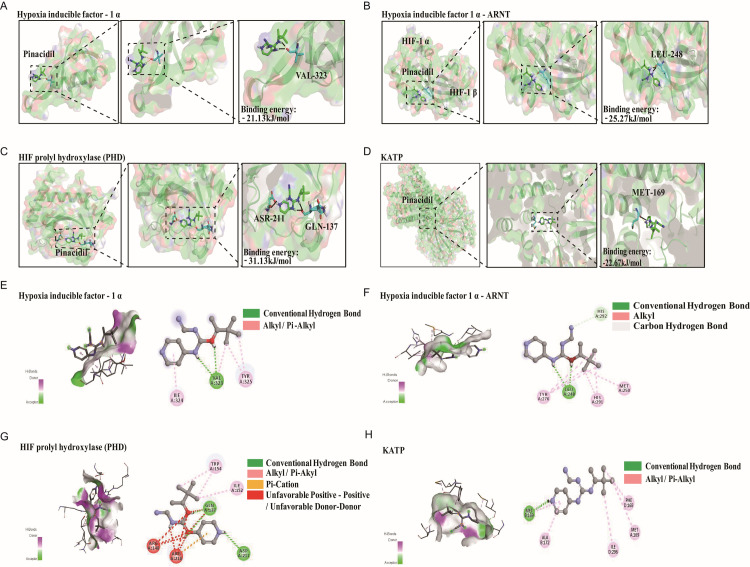
Computational simulation of molecular interaction between Pinacidil and HIF-1 **α,** **HIF-1α-ARNT, PHD or KATP.** (A-D) The representative interaction between Pinacidil and HIF-1α, HIF-1α-ARNT, PHD or KATP, respectively. (E-H) Rigid protein-protein docking images of Pinacidil with HIF-1α, HIF-1α-ARNT, PHD or KATP, respectively.

To further determine the binding site, we analyzed the docking process of Pinacidil with HIF-1α, HIF-1α-ARNT, PHD, and KATP. The results of Pinacidil binding with HIF-1α (Fig 6A and E) showed that the lowest binding free energy was -21.13 KJ/mol, there were two conventional hydrophobic bonds and one alkyl hydrophobic bond of HIF-1α as favorable structures donors interacting with Pinacidil. Among them, one of the active sites interacted with VAL323 (Valine located in sequence 323); one alkyl hydrophobic bond interacted with TYP 325 (Tryptophan in sequence 325); and the other Pi-Alkyl hydrophobic bond interacted with ILE 324 (Isoleucine in sequence 324) (Fig 6E).

Fig 6B and F showed that the lowest binding free energy of Pinacidil with HIF-1α-ARNT complex was −25.27 KJ/mol, there were two conventional hydrophobic bonds interacted with LEU248 (Leucine in sequence 248); and six alkyl interactions between TYR276 (Tyrosine in sequence 291), HIS291 (Histidine in sequence 291) and MET250 (Methionine in sequence 250); as well one Carbon hydrogen bond with HIS 292 in the advantageous structure (Fig 6F).

The results from Fig 6C and G indicated that the lowest binding free energy of Pinacidil with PHD was −31.13 KJ/mol, there were three conventional hydrophobic bonds interacted between GLN137 (Glutamine in sequence 137), ASP211 (aspartic acid in sequence 211) and Pinacidil; two active sites in the interaction with GLN137; three alkyl interactions between TRP154 and ILE152; eight unfavorable positive-positive bonds between GLN137, ARG148 (Arginine in sequence 137) and ARG 218, which contained two active sites (Fig 6G).

The results of binding of Pinacidil with KATP indicated that the lowest binding free energy was −22.67 KJ/mol (Fig 6D), one hydrophobic bond interacted with MET169; three hydrophobic bonds interacted with ILE296, MET169 and PHE168; two Pi-Alkyl bonds interacted with ALA 172 and MET169 were found in the structure (Fig 6H).

Based on the docking results, we speculated these hypotheses: the active sites of Pinacidil structure were focus on the two positive nitrogen of guanidine; the active sites of the relevant proteins structures could form strong binding bones (hydrogen bonds and unfavorable positive-positive bonds) with VAL, LEU, GLN, and ARG residues.

It has been reported that DMOG stabilizes and trans-activates HIF-1α, resulting in elevated endogenous levels of HIF-1α, leading to its accumulation in the cytoplasm and eventual transfer to the nucleus, where, together with HIF-1β responsible elements (HRE) to form HIF-1, mediates cardiomyocyte protection and participates in PHD [30]. Therefore, we used the molecular docking techniques to compare the pharmacological effects of Pinacidil and DMOG.

**Fig 7:**
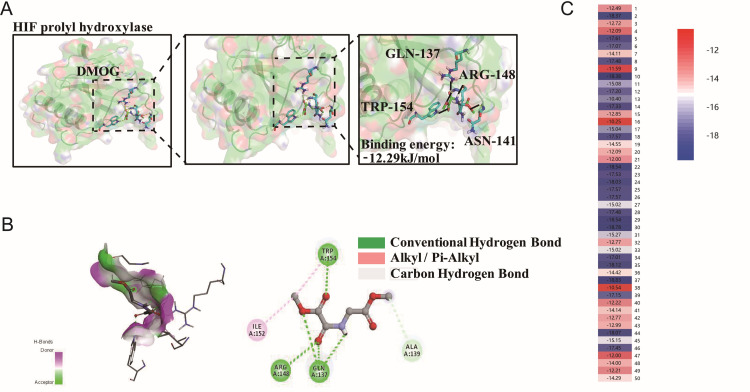
Results of docking of DMOG, HIF-1 **α** **prolyl hydroxylase, PHD**. (A) Docking structure of DMOG and HIF-1α prolyl hydroxylase; (B) Binding site of the complex; (C) Heatmap of docking results between DMOG and PHD.

The minimum binding energy of DMOG with HIF-1α prolyl hydroxylase was -12.19 kJ/mol, and there were five conventional hydrophobic bonds interacted between Pinacidil and TRP154, ARG148 or GLN137 (Fig 7A). Among them, there were three active sites: two alkyl interactions between TRP154 and ILE152; one carbon hydrogen bond between Pinacidil and ALA139 (alanine in sequence 139) (Fig 7B). “Fig 7B left” showed that DMOG tended to be donor rather than acceptor. The heatmap distribution indicated that there was a great tendency to interact with DMOG and PHD, the binding energy was concentrated in (−15 ~ −14) kJ/mol (Fig 7C).

These results suggested that Pinacidil has a lower binding potential and fewer active sites than DMOG. However, from our previous results, Pinacidil and DMOG have the similar therapeutic effects. These data suggested that Pinacidil has the ability to replace DMOG, exerting the protective effect on HR through PHD targets.

## Discussion

This study demonstrated the protective effect of Pinacidil on cardiomyocytes through *in vivo* and *in vitro* models of hypoxia or HR damage, and the mechanism of Pinacidil may be related to the protection of mitochondria by activating the HIF-1α/HRE pathway including the up-regulated expression of HIF-1α, iNOS, HO-1, VEGF. And we introduced the auto-docking and simulation analysis to predict the specific pharmacological mechanism by which Pinacidil binds to PHD and enhances its activity to up-regulate HIF-1α synthesis. The prediction of the guanidine active sites in Pinacidil and the high binding tendency of VAL, LEU, GLN, and ARG reveal potential targets of Pinacidil and IR damage, providing a new ideal for the design of cardiac protection studies.

Pinacidil, as a non-selective chemical K^ +^ channel openers [29], has a variety of cardioprotective effects [5,31,32]. KATP channel has powerful potential cardioprotective effect, KATP openers (KCOs) protect cardiomyocytes against ischemia and reperfusion injury by opening of KATP channels to trigger free radicals production and activate protein kinases. In this study, we investigated the cardioprotection of Pinacidil on HR cardiomyocytes and explored the mechanism.

According to that HIF-1α gene is up-regulated and largely bound binding to HRE under cardiac hypoxia conditions [33,34]. We tested the HIF-1α and others key proteins in the HIF-1α/HRE pathway, and found that Pinacidil significantly increased HIF-1α expression in cardiomyocytes after HR injury. The results from auto-docking predicted analysis predicted that Pinacidil up-regulated the HIF-1α protein expression by high active chemical interaction with PHD.

To detect the potential involvement targets of Pinacidil in cardiomyocytes HR injury, 5HD, MPG and DMOG were introduced in this study to block MitoKATP channel, scavenge ROS, and inhibit HIF prolyl hydroxylase to stabilize HIF-1α. Our results showed that no statistical difference in the mRNA expression of HIF-1α among the groups, however, the protein expression of HIF-1α was increased in HR group, and Pinacidil treatment largely enhanced the protein level of HIF-1α. HIF-1α dimerizes with HIF-1β/ARNT in the nucleus and then binds to HRE to form a complex that activates transcription of target genes in response to oxidative stress [35]. VEGF-A, iNOS, and HO-1, as target genes of HIF-1α/HRE pathway, are highly correlated with HR cardiomyocytes injury. Therefore, we further studied the mRNA and the protein expressions of VEGF-A, iNOS and HO-1. The results showed that the mRNA and the protein expression of VEGF, iNOS, HO-1 were significantly increased in HR cardiomyocytes, Pinacidil greatly enhanced HIF-1α protein expression and promoted HRE to target VEGF, iNOS and HO-1 in HR model. The results suggested that Pinacidil may act as a potential modulator of HIF-1α to regulate the downstream targets expression.

We used auto-docking and Discovery Studio visualizer analysis in this study to predict the possible pharmacological sites of Pinacidil with HIF-1α, ANRT, HIF-1α-ANRT complex, VEGF-A, iNOS, HO-1, PHD and KATP channel protein. And the results showed that Pinacidil has the highest binding trend with PHD, even higher than DMOG, indicating that PHD is the most possible target of Pinacidil to up-regulate HIF-1α. The chemical structure of Pinacidil analysis points out that the active sites concentrated on the 2 positive-nitrogen groups on guanidine are key pharmacological groups for binding to PHD (Fig 6), and their binding capacity may be superior to that of DMOG (Fig 7). Based on these results, Pinacidil is a safer clinical drug compared to DMOG. Our study reveals a new potential drug for the treatment of IR patients and predicts the chemical synthesis of a meaningful IR drug development.

This founding provides a possible mechanistic explanation for the link between HIF-1α and the adverse prognosis in IR patients. Considering that Pinacidil can modulate HIF-1α expression in our study, it is plausible to speculate that the observed effects of Pinacidil on myocardial ischemia-reperfusion injury may be mediated through a possible interaction with HIF-1α, and subsequent regulation this signaling pathway. Further studies are needed to validate and expand upon these findings, paving the way for potential clinical applications of Pinacidil in the management of myocardial ischemia-reperfusion injury condition.
